# Selection is more intelligent than design: improving the affinity of a bivalent ligand through directed evolution

**DOI:** 10.1093/nar/gks899

**Published:** 2012-10-05

**Authors:** Kareem M. Ahmad, Yi Xiao, H. Tom Soh

**Affiliations:** ^1^Interdepartmental Program in Biomolecular Science and Engineering, ^2^Department of Mechanical Engineering and ^3^Department of Materials, University of California Santa Barbara, Santa Barbara, CA 91306, USA

## Abstract

Multivalent molecular interactions can be exploited to dramatically enhance the performance of an affinity reagent. The enhancement in affinity and specificity achieved with a multivalent construct depends critically on the effectiveness of the scaffold that joins the ligands, as this determines their positions and orientations with respect to the target molecule. Currently, no generalizable design rules exist for construction of an optimal multivalent ligand for targets with known structures, and the design challenge remains an insurmountable obstacle for the large number of proteins whose structures are not known. As an alternative to such design-based strategies, we report here a directed evolution-based method for generating optimal bivalent aptamers. To demonstrate this approach, we fused two thrombin aptamers with a randomized DNA sequence and used a microfluidic *in vitro* selection strategy to isolate scaffolds with exceptionally high affinities. Within five rounds of selection, we generated a bivalent aptamer that binds thrombin with an apparent dissociation constant (K_d_) <10 pM, representing a ∼200-fold improvement in binding affinity over the monomeric aptamers and a ∼15-fold improvement over the best designed bivalent construct. The process described here can be used to produce high-affinity multivalent aptamers and could potentially be adapted to other classes of biomolecules.

## INTRODUCTION

Multivalency offers a powerful mechanism for dramatically increasing the affinity and specificity of molecular interactions. To choose an example from nature, tetraubiquitin chains exhibit a ∼700-fold increase in affinity for the TAK1 protein (a protein kinase of the MLK family) relative to the weak interactions observed for single ubiquitin molecules, and this differential binding affects critical cellular functions such as transcriptional regulation and cell survival and proliferation (1). Multivalent molecular interactions have also yielded marked improvements in the efficacy of antibodies developed for biological therapies (2) and engineered glycoclusters (3). Several groups have shown that the affinity and specificity of nucleic acid-based aptamers (4,5) can also be enhanced through multivalency (6). For example, Müller *et al.* (7) were the first to demonstrate that two aptamers that bind to distinct epitopes on the protein thrombin could be linked together to improve overall affinity. They used a flexible 15-nucleotide (nt) poly-adenine (poly-A) linker to connect the two aptamers, and demonstrated a ∼3-fold improvement in affinity over the monovalent molecules. Subsequently, Rinker *et al.* (8) used a rigid DNA scaffold as a linker and reported an even greater (50-fold) improvement in affinity using the same set of aptamers. Finally, Tian and coworkers (9) reported a bivalent construct using these same two thrombin aptamers and a flexible linker with a 97-fold affinity improvement.

We believe that one of the main sources of this variability lies in the design of the scaffold joining the two aptamers. An ideal scaffold must place the two aptamers at optimal positions and orientations with respect to their epitopes on the target molecule, and must not interfere with the aptamer structures. The rational design of linkers that fulfil these requirements is a challenging problem for proteins with known structures, and remains an insurmountable obstacle for the large number of proteins whose structure is unknown.

In this work, we demonstrate a novel alternate strategy to address this problem. Instead of using rational design-based methods, we have used a directed evolution strategy to select for a bivalent aptamer with a high-affinity scaffold. After five rounds of microfluidic selection, we isolated and characterized a bivalent aptamer that binds thrombin with an apparent dissociation constant (K_d_) of 8.1 pM: a ∼200-fold improvement in affinity over either of the two parental aptamers alone, and significantly better than the best rationally designed DNA constructs described to date (7–10).

## MATERIALS AND METHODS

### DNA and target preparation

The single-stranded DNA (ssDNA) library and (poly-thymine) poly-T substituted sequences were purchased from Integrated DNA Technologies. Labeled polymerase chain reaction (PCR) primers and designed bivalent aptamers were obtained from Biosearch Technologies with reverse-phase high pressure (or high performance) liquid chromatography purification. Human α-thrombin was purchased from Haematologic Technologies. The process of protein immobilization on magnetic beads (M-270, carboxylic acid functionalized, Life Technologies) was performed according to the manufacturer’s protocol for two-step coupling using ethyl (dimethylaminopropyl) carbodiimide and *N*-hydroxysuccinimide (Sigma). Surface coverage of the immobilized protein was estimated using the NanoOrange Protein Quantitation kit (Life Technologies), following the manufacturer’s protocol. The amount of protein coupled was calculated after subtracting the fluorescence intensity of Tris-blocked beads from the fluorescence of the protein-coupled beads, and verified by measuring the remaining uncoupled protein in the supernatant at the conclusion of the reaction.

### Device fabrication

The micromagnetic separation (MMS) chip was fabricated and assembled according to a process previously described by our group (11–13). Briefly, the device was fabricated on borosilicate glass substrates with 25 -µm-thick double-coated tape (3M). The micropattern was defined on the bottom glass substrate with 20-nm-thick titanium and 200-nm-thick nickel films using standard photolithography methods. Pitch distances of 200, 100 and 50 µm were used in the nickel grid pattern to provide increasing grid density. Fluidic vias were drilled through the glass substrates using a computer-controlled CNC mill and diamond bit (Abrasive Technology). After cleaning with acetone, the Ti/Ni layer was passivated with a 100-nm-thick layer of SiO_2_ by plasma-enhanced chemical vapor deposition (Plasma-Therm). Microfluidic channels were cut out of the 25 -µm-thick double-sided tape using a plotting cutter (Graphtec). The patterned tape was overlaid onto the top glass substrate manually. The channel and micropattern were then manually aligned, and the device was cured in an oven at 70°C overnight with light clamping pressure. A brass eyelet was used as the buffer inlet, and Tygon tubing (Saint-Gobain) was used for the sample inlet and outlet fluidic connections. All connections were glued in place using 5-min epoxy (Devcon). The external magnets consisted of eight stacked neodymium magnets (K&J Magnetics) with another magnet of the same type sandwiching the device to secure it during the separation and washing steps.

### Microfluidic aptamer selection

The selection buffer used for incubation and washing consisted of 50 mM Tris, 100 mM NaCl, 1 mM MgCl_2_, 5 mM KCl, 1 mM CaCl_2_ and 0.01% Tween 20 at pH 7.5. Selection conditions are summarized in Supplementary Table S1. DNA was incubated with thrombin-coated beads for 1 h. All washes were performed at a flow rate of 50 ml/h. After microfluidic washing, we eluted the beads in 100 µl of buffer and amplified 10 µl of eluate by pilot PCR to determine the optimal number of cycles required for amplification of the remaining eluate without accumulation of amplification byproduct. The eluted DNA was then PCR amplified using a phosphorylated reverse primer and fluorescein amidite-labeled forward primer. This double-stranded DNA was purified with Qiagen minElute columns, and the concentration was measured by ultraviolet spectroscopy. Finally, we generated ssDNA by using lambda exonuclease (New England Biolabs) to digest the 5′-phosphorylated reverse strand. The ssDNA was phenol–chloroform extracted, then ethanol precipitated and finally resuspended in 20 µl of ultrapure water. The concentration was measured by ultraviolet spectroscopy, and the purity was verified on a 4.5% low-melt agarose gel prestained with GelStar (Lonza). Cloning and sequencing were performed as described previously (12) using the TOPO TA Cloning Kit (Life Technologies). Individual bacterial clones and selected sequences were directly PCR amplified using modified primers, and then processed as aforementioned to obtain purified fluorescently labeled ssDNA for subsequent binding assays.

### Aptamer binding assays

To measure the binding affinities of the selected pools and sequences, we incubated a range of concentrations of fluorescein amidite-labeled ssDNA with target-coated beads for 1 h at room temperature with gentle rotation. Each sample was then washed four times using a Magnetic Particle Concentrator (Life Technologies), and the remaining bound DNA was eluted by incubation in selection buffer at 95°C for 10 min. The amount of fluorescence in the supernatant (RFU) was measured with a microplate reader (Tecan), and the background subtracted fluorescence values were fit to a saturation binding curve [RFU = [ssDNA]*RFU_max_/([ssDNA]+K_d_)] using nonlinear regression (assuming 1:1 binding) with Origin software (OriginLab). Because we use washing and eluting steps, our binding measurements are not strictly performed in equilibrium.

### Thrombin inhibition assays

Thrombin inhibition assays were performed by monitoring the formation of insoluble fibrin by measuring the absorbance of the reaction at 350 nm on a microplate reader (Tecan) at 25°C. Experiments to determine K_m_ were performed with 1 nM of thrombin and a range of fibrinogen concentrations in selection buffer at a reaction volume of 150 µl. To determine the half-maximal inhibitory concentrations (IC_50_s) of the thrombin inhibitors, we prepared reactions containing 0.04 nM thrombin, 2 µM fibrinogen (Sigma) and various inhibitors at a range of different concentrations in 300 µl of selection buffer in duplicate. For each thrombin inhibitor, the absorbances at steady state were blank subtracted then normalized. These values were fitted to a four-parameter logistic model (Origin) by nonlinear regression to calculate the IC_50_. The model takes the form: y = D + (A − D)/(1 + (x/C)^B^, where y = normalized absorbance, x = the inhibitor concentration, and with the four parameters, A = absorbance with no inhibition, B = the slope factor, C = IC_50_ and D = absorbance at maximum inhibition (14). The inhibitory constants (K_i_) were calculated from the measured K_m_ of the uninhibited reaction and the measured IC_50_s using the Cheng–Prusoff equation. Argatroban was obtained from Sigma and dissolved in selection buffer.

### Aptamer melting temperatures

Melting curves were obtained with 200 ng of DNA and a 1× concentration of SYBR Green in 20 µl of selection buffer using a real-time thermal cycler (Bio-Rad), with temperatures ramped from 20 to 95°C at a rate of 2°C/min. Melting temperatures (T_m_) were calculated by the CFX Manager software (Bio-Rad).

## RESULTS AND DISCUSSION

### Directed evolution of bivalent aptamers

**Figure 1.**
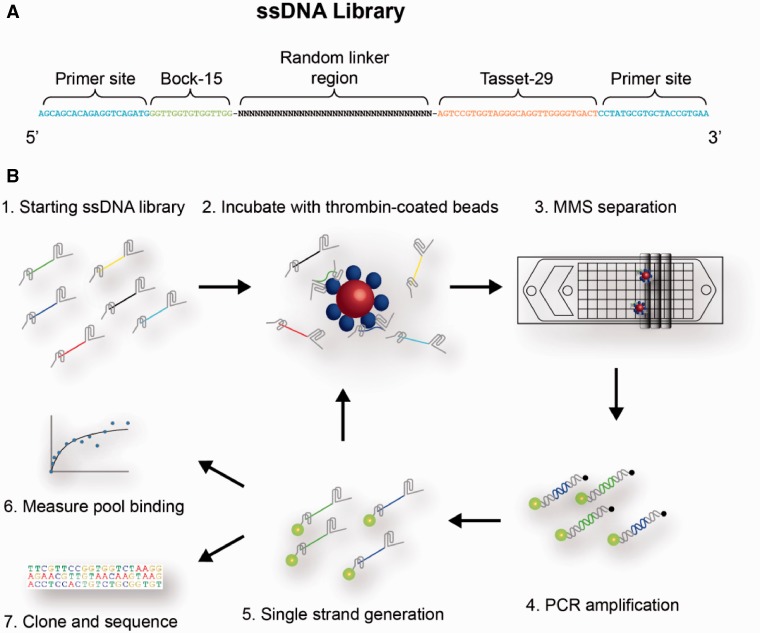
Overview of the bivalent aptamer linker selection process. (**A**). Each molecule in the ssDNA library consists of two thrombin aptamers, Bock-15 and Tasset-29, joined by a 35-nt randomized linker region and flanked by two primer sites for PCR amplification. (**B**) The major steps of the microfluidic selection process are shown. Bivalent aptamers are captured by binding to thrombin-coated magnetic beads, and then washed within the MMS device to eliminate weakly and nonspecifically bound ssDNA. The resulting pool is then PCR amplified in a manner that enables rapid subsequent preparation of single-stranded molecules, which can then be subjected to further rounds of selection or directly characterized based on sequence and binding affinity.

We constructed a combinatorial library of 119-nt ssDNA sequences, each of which contains two previously published thrombin-binding aptamers: Bock-15 (15), which binds the fibrinogen-binding exosite, and Tasset-29 (16), which targets the heparin-binding exosite (Figure 1A). These are the same two aptamers that were used in previous studies of bivalency (7–10). The central linker region connecting these two aptamers consists of a 35-nt tract of randomized bases. We chose this length so that the linker region easily spans the distance between the two binding sites (2.5 nm) and contains sufficient nucleotides to fold into an optimal structure that supports interaction with the target molecule. Each library member is flanked by additional primer sites at both ends for PCR amplification.

Starting with this library, we performed five rounds of selection using the MMS device previously described by our group (11,12) (Figure 1B). The MMS device enables highly stringent selection conditions by allowing the use of low concentrations of protein during the incubation and continuous washing at high flow rates (50 ml/h) for extended periods (20 min) without the loss of beads or bound aptamers. These features enforce the efficient removal of weakly bound molecules from the target, which accelerates the isolation of aptamers with high affinity (13). The conditions used in each round of selection are shown in Supplementary Table S1.

**Figure 2.**
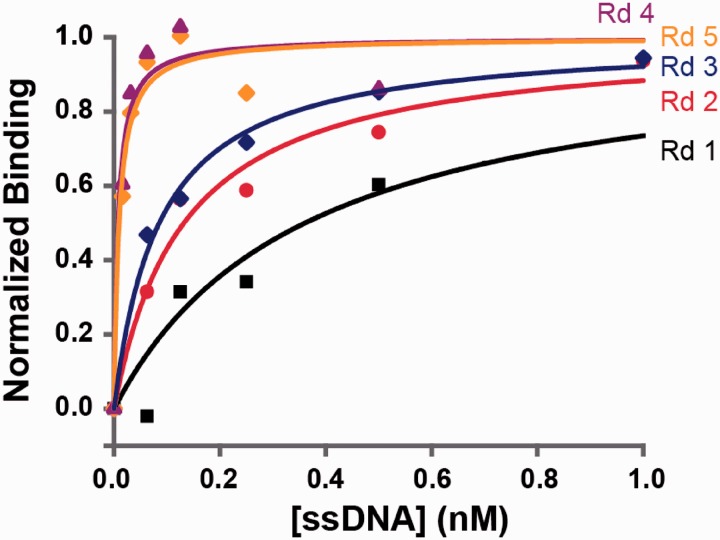
Binding affinities of enriched aptamer pools for 5 rounds of selection. Measurements for each round are based on background-subtracted fluorescence values from a bead-based binding assay, scaled to saturation intensity. Binding affinity against thrombin increased over four rounds of selection, from an apparent K_d_ of 0.361 nM in Round 1 to 0.0075 nM in Round 4, before plateauing in Round 5.

We measured the bulk affinities of the enriched pools after each round using a bead-based fluorescence assay (12), and observed that the affinity increased monotonically until Round 4 before plateauing in Round 5 (Figure 2). The measured affinities of the pools were 0.361 nM for Round 1, 0.132 nM for Round 2, 0.085 nM for Round 3, 0.0075 nM for Round 4 and 0.0094 nM for Round 5.

### Selected bivalent aptamer binds with high affinity

**Figure 3.**
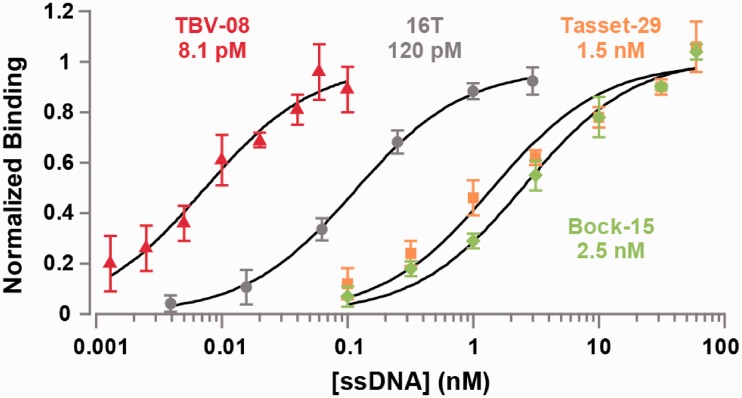
Directed evolution of an aptamer linker sequence yields significant gains in binding affinity. Normalized mean fluorescence intensities from aptamer binding assays performed in triplicate with thrombin-coated beads are shown, along with standard deviations. Our highest-affinity selected sequence (TBV-08) binds much more strongly than the parental aptamers (Bock-15 and Tasset-29) and a bivalent thrombin aptamer joined by a poly-T linker (16T). Measured apparent disassociation constants (K_d_s) are shown below labels.

We cloned the Round 5 pool and randomly picked 10 clones for sequencing. Although these sequences contained no obvious consensus (Supplementary Table S2), all 10 showed strong affinity for thrombin based on binding measurements with 50 pM aptamer (Supplementary Figure S1). We synthesized the top binding sequence (TBV-08), fluorescently labeled it with fluorescein and measured its affinity for thrombin. TBV-08 exhibited an apparent K_d_ of 8.1 pM, an affinity ∼200-fold higher than that of parental aptamer Tasset-29 and ∼300-fold higher than Bock-15 (Figure 3).

The affinity enhancement of TBV-08 also outperforms the best bivalent aptamer constructs described to date. By comparison, using their methods and conditions, Hasegawa *et al.* (10) reported an improvement of 58-fold using a flexible poly-T linker, whereas Tian *et al.,* reported a K_d_ improvement of 97-fold with a flexible PEG linker. We benchmarked TBV-08 by testing the binding affinities of a large group of bivalent aptamers incorporating flexible poly-T linkers of varying length (Supplementary Figure S2). Of these, the construct with the 16T linker exhibited the highest affinity (apparent K_d_ = 120 pM), in reasonable agreement with previous studies (9,10). However, TBV-08 binds 15-fold better than this construct (Figure 3).

Next, we investigated the basis for this affinity enhancement. We measured the affinities of modified versions of TBV-08 in which we substituted either one or both of the individual aptamers with poly-T tracts of equivalent length, enabling us to examine each aptamer’s contribution to binding within the context of the whole sequence. We also measured the affinity of the selected linker sequence itself, without the two parental aptamers. When both aptamers were substituted with poly-T tracts, the resulting sequence had poor (>300 nM) affinity for thrombin (data not shown). This excludes the possibility that the linker is folding into a third aptamer and interacting with the protein directly. For the single aptamer substitutions, the affinity of the Tasset-29 aptamer plus selected linker was somewhat worse than that of the aptamer in isolation, whereas that of the Bock-15 aptamer was slightly improved (Supplementary Figure S3).

These results indicate that the affinity enhancement is not due to improvements in the individual affinities of either of the two aptamers, nor to direct interactions between the scaffold and the protein. Instead, we speculate that this enhancement appears to arise through scaffold-mediated reorientation of the two aptamers into optimal positions relative to their respective binding sites, which presumably reduces the entropy loss that occurs on binding (17).

To better understand the mechanism of binding enhancement, we explored the quantitative structure–activity relationship of the aptamer sequences and their relative affinities. From scatter plots of the relative binding affinity of each aptamer versus the proportion of each base, it appears that the affinity is dependent on the A base fraction (*r*^2^ = 0.51) and weakly inversely related to the G base fraction (*r*^2 ^= 0.22) (Supplementary Figure S4A). The increase in relative affinity with the base fraction of A’s may be because of favorable base stacking (18). Although similar base stacking will occur with G’s as well, a high fraction of G’s in the selected sequence is disfavored probably because it will interfere with quadruplex formation in the parental aptamers.

One possible explanation for the high affinity in the selected aptamers is that rigid structural elements in the scaffold favorably align the parental aptamers with their binding sites on thrombin. This rigidity is afforded by extensive base pairing in the scaffold, which constrains potential alternate aptamer conformations. To test this hypothesis that more rigidity leads to better binding affinity, we plotted the number of base pairs in the scaffold, as a proxy for rigidity, versus the relative binding affinity and found the two properties to be unrelated (Supplementary Figure S4B). To obtain a more sophisticated estimate of structural stability, we used mfold (19) to estimate the ΔG’s of the lowest free energy structures from the aptamer sequences. Plotting this estimate versus the relative binding affinity yielded the same result (Supplementary Figure S4C), namely, that binding affinity is independent of structural stability. This suggests that binding affinity is not a simple dichotomy between flexibility and rigidity. Indeed, it is precisely this subtlety and complexity in molecular interactions that motivated us to pursue a directed evolution strategy in lieu of a design approach based on first principles.

### Selected bivalent aptamer effectively inhibits thrombin

**Figure 4.**
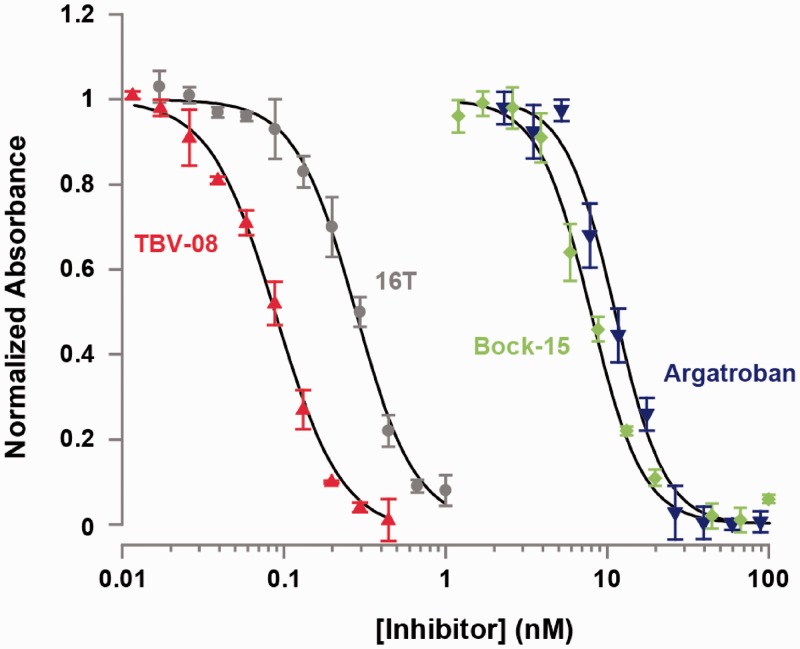
The selected TBV-08 aptamer markedly improves inhibition of thrombin activity. We plotted the absorbance (OD_350_) of the thrombin-mediated fibrinogen cleavage reaction at steady state in the presence of varying concentrations of different inhibitory molecules. These data were fitted to a four-parameter logistic model and scaled from 0 to 1. TBV-08 proved more effective than the direct thrombin inhibitor Argatroban, the parental aptamer Bock-15 and the 16T bivalent aptamer.

In addition to binding thrombin with higher affinity, TBV-08 is more effective at inhibiting thrombin activity. Thrombin, which cleaves the protein fibrinogen, is the key protease responsible for the final step in blood coagulation (20). The Bock-15 aptamer inhibits this process by interacting with thrombin’s fibrinogen binding site (21). To show that TBV-08 is a more effective inhibitor, we assessed its effects on thrombin-catalyzed formation of insoluble fibrin relative to Bock-15, the 16T bivalent aptamer, and Argatroban, a small-molecule inhibitor of thrombin (Figure 4) (20).

By monitoring the absorbance of the reaction at 350 nm, we measured the amount of insoluble fibrin produced and thereby calculated the IC_50_ of each molecule. We calculated inhibitory constants (K_i_) using the measured K_m_ for the reaction (Supplementary Figure S5), and the IC_50_ for inhibitors using the Cheng–Prusoff equation. These data show that TBV-08 inhibits thrombin >3-fold better than the 16T bivalent aptamer (Table 1). Such an improvement in inhibition of enzymatic activity was shown previously for the case of a bivalent aptamer, which binds DNA polymerase (22).

**Table 1.** Quantification of thrombin inhibition by different molecules

Thrombin inhibitor	IC_50_ (nM)	K_i_ (nM)
Argatroban	11.2	3.7
Bock-15	7.9	2.61
16T bivalent aptamer	0.280	0.092
TBV-08	0.089	0.030

We performed thrombin-catalyzed conversion of fibrinogen to fibrin in the presence of various concentrations of Bock-15 aptamer, 16T bivalent aptamer, TBV-08 and the small-molecule inhibitor Argatroban, and calculated the K_i_ from the measured IC_50_ for each inhibitor. Based on these data, TBV-08 was by far the most effective thrombin inhibitor tested.

### Selected bivalent aptamers exhibit structural similarities

**Figure 5.**
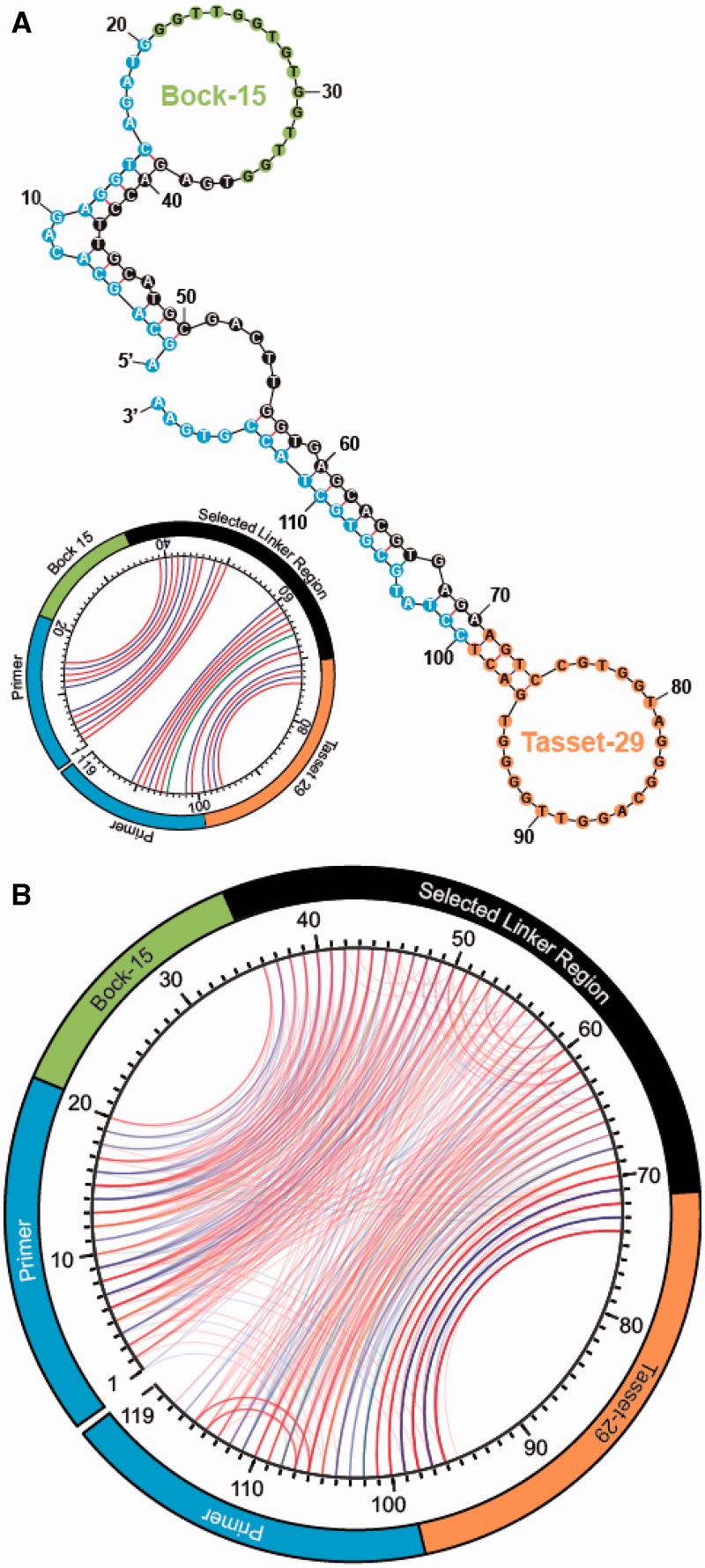
Predicted secondary structures of TBV-08 and other high-affinity sequences from Round 5. (**A**) The lowest free energy computed Watson–Crick secondary structure predicted for TBV-08. PCR primer sites are highlighted in blue, the selected linker region in black and parental thrombin aptamers in green (Bock-15) and orange (Tasset-29). The secondary structure of TBV-08 is also shown as a circle plot (inset). Lines connect positions that are base paired (red = G-C, blue = A-T and green = wobble). G-quartet interactions are not shown. The primers sites show extensive base pairing with the selected linker region. (**B**) The secondary structures of all 29 Round 5 sequences are overlaid as circle plots. Even in the absence of a clear consensus sequence, the aptamer secondary structures show substantial internal symmetry and homology with one another, demonstrating clearly, the effects of selection.

To better understand the structural basis for the higher affinity, we used the mfold application (19) to visualize the predicted Watson–Crick base pairing of the best binding aptamer sequence, TBV-08. In its lowest free energy structure, TBV-08 forms an elongated dumbbell, with the two thrombin aptamers forming loops at either end (Figure 5A). These two domains are joined by a short single-stranded hinge region that would allow the two aptamers to pivot and independently orient themselves for optimal binding. Unexpectedly, we also observe that the primer sites play an important role by interacting with the selected linker region to form rigid double-helical stems. Thus, a combination of some structural rigidity and conformational flexibility appears to provide the best affinity.

To further investigate the nature of the linkers, we obtained 19 additional clones and modeled their secondary structures using mfold (19). All 29 structures are shown in Supplementary Figure S6. Although every sequence was unique, the structures predicted to have the lowest free energy state share some common features. For example, the primer sites consistently form stems with the selected region, and in most cases, there is a central, single stranded, ‘hinge’ region. We constrained the predicted models by specifying that the two G-quadruplexes in the parental aptamers (16) should remain unpaired, but unconstrained predictions yielded identical structures in >50% of sequences.

We also visualized the predicted secondary structure via an alternate method known as a circle plot (19), wherein the entire sequence is laid out on the edge of a circle, and paired bases are connected by line segments (Figure 5A, inset). When overlaid, the circle plots for all 29 sequences from Round 5 reveal remarkable similarity at the structural level (Figure 5B) despite the lack of conservation at the sequence level (Supplementary Table S2 and Figure S6), demonstrating the potent effects of selection in driving convergence toward a physical conformation that favors bivalent interaction. As predicted for TBV-08, the majority of these structures feature substantial base pairing between the primer sites and proximal segments of the selected linker region.

To confirm the contributions of linker–primer site interactions to aptamer structure and function, we performed two sets of corroborative experiments. First, binding assays with a truncated version of TBV-08 lacking primer sites exhibited dramatically reduced binding affinity—from 8.1 to 440 pM—as would be expected if these sequences were crucial components of the aptamer’s secondary and tertiary structure (Supplementary Figure S7). Next, we observed that the melting temperature of TBV-08 was substantially higher than the melting temperatures of the parental aptamers and 16T bivalent aptamer, which demonstrated that the primer sites and selected linker sequence confer considerably enhanced structural stability consistent with the predicted model (Supplementary Table S3).

## CONCLUSION

We report a directed evolution-based strategy to select bivalent aptamers with a scaffold that considerably improves affinity. This strategy for the control of biomolecules requires no *a priori* structural or chemical information except for the knowledge that the binding sites for the two ligands are nonoverlapping. As a demonstration, we isolated a bivalent aptamer against thrombin with an apparent K_d_ of 8.1 pM, a ∼200-fold improvement over the monomeric aptamers and a ∼15-fold improvement over the best rationally designed DNA constructs. We further show that this improvement in binding affinity translates into more effective thrombin inhibition.

The combination of structural rigidity and flexibility conferred by the scaffold in these high-affinity structures would be extremely difficult to reproduce using a design-based strategy (8,17). Interestingly, nature has evolved a similar solution to this problem, hirudin—the anticoagulant peptide produced by leeches binds to thrombin with high affinity and specificity through a combination of rigid and flexible structural elements, as confirmed by computational modeling (23). Extending our method of bivalent scaffold selection to incorporate peptides and proteins may be feasible because our selection strategy for generating a high-affinity nucleic acid scaffold does not rely on specific chemical knowledge of the molecular structure of the target molecule.

## SUPPLEMENTARY DATA

Supplementary Data are available at NAR Online: Supplementary Tables 1–3 and Supplementary Figures 1–7.

## FUNDING

Army Research Office through the Institute for Collaborative Biotechnologies [W911NF-09D0001, W911NF-10-2-0114]; National Institutes of Health (NIH) [U54DK093467, R01EB009764]. Funding for open access charge: NIH [R01EB009764].

*Conflict of interest statement*. None declared.

## Supplementary Material

Supplementary Data

Supplementary Data
