# Supplementary material for: Selection is more intelligent than design: improving the affinity of a bivalent ligand through directed evolution
Source: Nucleic Acids Res. 2012 Oct 5;40(22):11777–83. doi: 10.1093/nar/gks899 (PMC3526301; doi:10.1093/nar/gks899)
Supplement: Supplementary Data [file supp_gks899_nar-01831-met-f-2012-File002.docx]

**SUPPLEMENTARY DATA**

Supplementary Data are available at NAR online: Supplementary Tables 1-3, Supplementary Figures 1-7.

| **Parameter** | **Round** | | | | |
| --- | --- | --- | --- | --- | --- |
|  | **1** | **2** | **3** | **4** | **5** |
| DNA quantity (pmol) | 1,000 | 5 | 1 | 0.2 | 0.1 |
| DNA quantity (molecules) | 6.0*10^14^ | 3.0*10^12^ | 6.0*10^11^ | 1.2*10^11^ | 6.0*10^10^ |
| Incubation volume (µL) | 5,000 | 5,000 | 5,000 | 5,000 | 5,000 |
| DNA concentration (pM) | 200,000 | 1,000 | 200 | 40 | 20 |
| Protein concentration (pM) | 20 | 20 | 20 | 10 | 10 |
| Number of beads | 2*10^5^ | 2*10^5^ | 2*10^5^ | 1*10^5^ | 1*10^5^ |

**Table S1.** Summary of selection conditions for each round.

| **Clone** | | **Sequence** |
| --- | --- | --- |
| 1 | AGCAGCACAGAGGTCAGATGGGTTGGTGTGGTTGG**AGCGGACTTTACCTGTGTCAGCTGCGGGGGCCGCA**AGTCCGTGGTAGGGCAGGTTGGGGTGACTCCTATGCGTGCTACCGTGAA | |
| 2 | AGCAGCACAGAGGTCAGATGGGTTGGTGTGGTTGG**CTGATACCGGAAGGGGTGTGTGTAGCGCGGTTGAG**AGTCCGTGGTAGGGCAGGTTGGGGTGACTCCTATGCGTGCTACCGTGAA | |
| 3 | AGCAGCACAGAGGTCAGATGGGTTGGTGTGGTTGG**TGAGTCGACTGTGTGCCTCAAGTAGAACGTAGGTG**AGTCCGTGGTAGGGCAGGTTGGGGTGACTCCTATGCGTGCTACCGTGAA | |
| 4 | AGCAGCACAGAGGTCAGATGGGTTGGTGTGGTTGG**CTGGTCTGGGGGCGTTTACTGGGCGCGCATTTTAA**AGTCCGTGGTAGGGCAGGTTGGGGTGACTCCTATGCGTGCTACCGTGAA | |
| 5 | AGCAGCACAGAGGTCAGATGGGTTGGTGTGGTTGG**CATTTTGACCGAATGACGTTAGCAAAATATGTTAG**AGTCCGTGGTAGGGCAGGTTGGGGTGACTCCTATGCGTGCTACCGTGAA | |
| 6 | AGCAGCACAGAGGTCAGATGGGTTGGTGTGGTTGG**CGAGGACGCTGTGTGCGCTTCGAGGGCCTAGAAGG**AGTCCGTGGTAGGGCAGGTTGGGGTGACTCCTATGCGTGCTACCGTGAA | |
| 7 | AGCAGCACAGAGGTCAGATGGGTTGGTGTGGTTGG**CTCTGATAACCCGGGGCTGTGGTGCGCCAGGTAGG**AGTCCGTGGTAGGGCAGGTTGGGGTGACTCCTATGCGTGCTACCGTGAA | |
| 8 | AGCAGCACAGAGGTCAGATGGGTTGGTGTGGTTGG**TGAGACCTTGCATGCGACTTGGTGAGCACGTGAGA**AGTCCGTGGTAGGGCAGGTTGGGGTGACTCCTATGCGTGCTACCGTGAA | |
| 9 | AGCAGCACAGAGGTCAGATGGGTTGGTGTGGTTGG**CATTCGTAACTATGGGGTTCCTGTATTGGTATAGG**AGTCCGTGGTAGGGCAGGTTGGGGTGACTCCTATGCGTGCTACCGTGAA | |
| 10 | AGCAGCACAGAGGTCAGATGGGTTGGTGTGGTTGG**TTTGCCGATGTGTGGACAGCATGTGGGGTAGAGCG**AGTCCGTGGTAGGGCAGGTTGGGGTGACTCCTATGCGTGCTACCGTGAA | |

**Table S2.** Ten aptamer sequences obtained from the round 5 pool. Full sequences are shown 5’ to 3’. Blue = primer sites, green = Bock-15, orange = Tasset-29, and black = Selected region. All sequences were unique, and no obvious homology was evident.

| **Sequence** | **T_m_ (°C)** |
| --- | --- |
| Bock-15 | 32.0 |
| Tasset-29 | 35.0 |
| 16T bivalent aptamer | 35.0 |
| TBV-08 | 60.0 |

**Table S3.** Sequence features of TBV-08 confer considerable additional structural stability. We measured the melting temperatures (T_m_) of each parental aptamer, the 16T bivalent aptamer and TBV-08. The T_m_ of TBV-08 was far higher than for the other molecules, indicating a greater degree of stability that is most likely due to extensive hybridization between the primer site and the selected linker region.


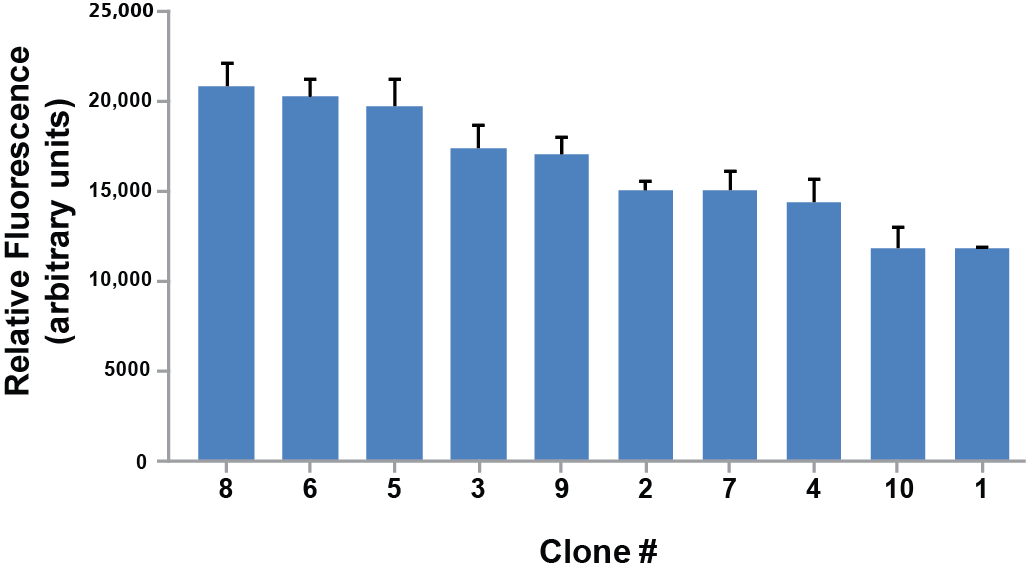


**Figure S1.** The relative binding affinities of 10 clones from the round 5 pool were measured with a bead-based fluorescence assay at 50 pM of aptamer and ranked in descending order. Mean fluorescence values and standard deviations of two measurements for each clone are shown. Clone # 8 (TBV-08) exhibited the highest relative affinity.

**
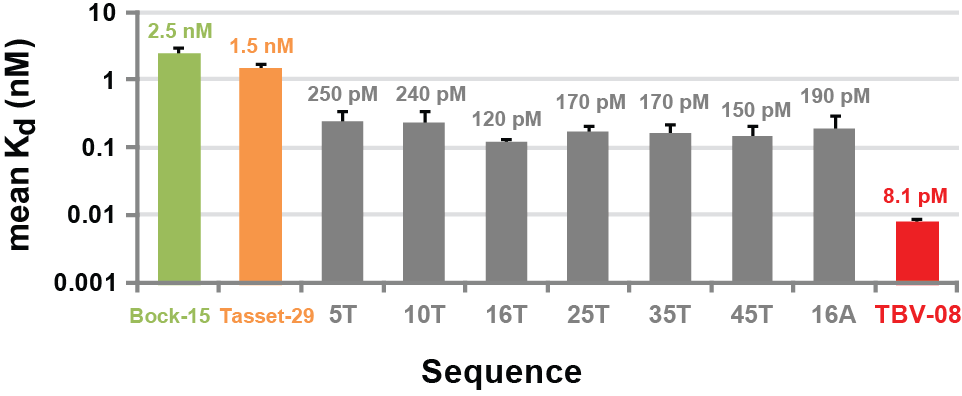
**

**Figure S2.** Comparison of binding affinities of bivalent aptamers with different linker lengths. The labeled mean apparent K_d_s and standard deviations are based on three independent measurements. The selected bivalent aptamer sequence (TBV-08) binds with significantly higher affinity than the individual parental thrombin aptamers (green and orange) as well as bivalent aptamers with poly-T and poly-A linkers of varying lengths.


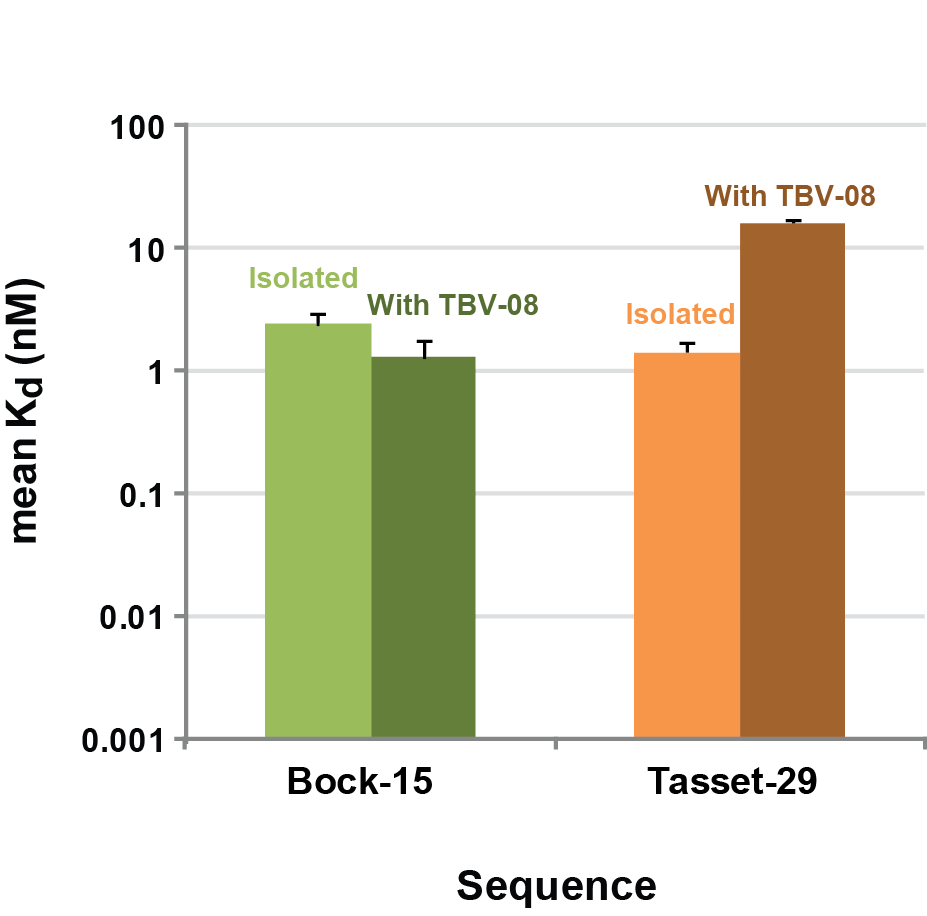


**Figure S3.** Apparent binding affinities of modified TBV-08 sequences measured with a bead-based fluorescence assay. The affinities of the parental thrombin aptamers do not substantially improve in the context of the selected sequence.

**
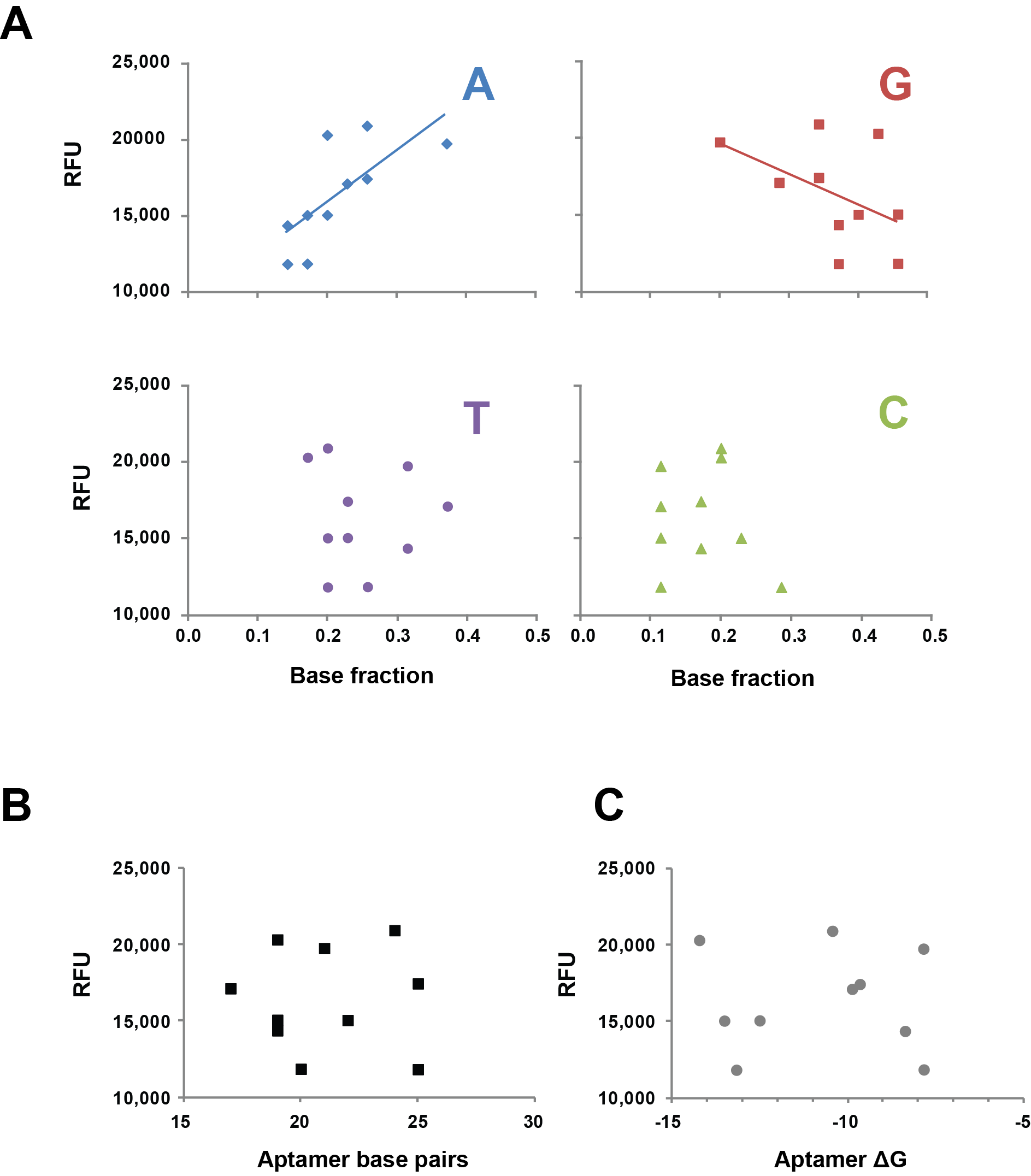
**

**Figure S4.** Quantitative structure-activity relationship. (A) Scatter plots of relative binding affinities of each sequence (RFU) from Fig S1 plotted against the base fractions of each of the four bases. Binding is related to fraction of As and inversely related to the fraction of Gs and is independent of the fraction of Ts and Cs. Also shown, are scatter plots demonstrating no dependence of relative binding affinity on number of aptamer base pairs (B) or aptamer ∆G (C).


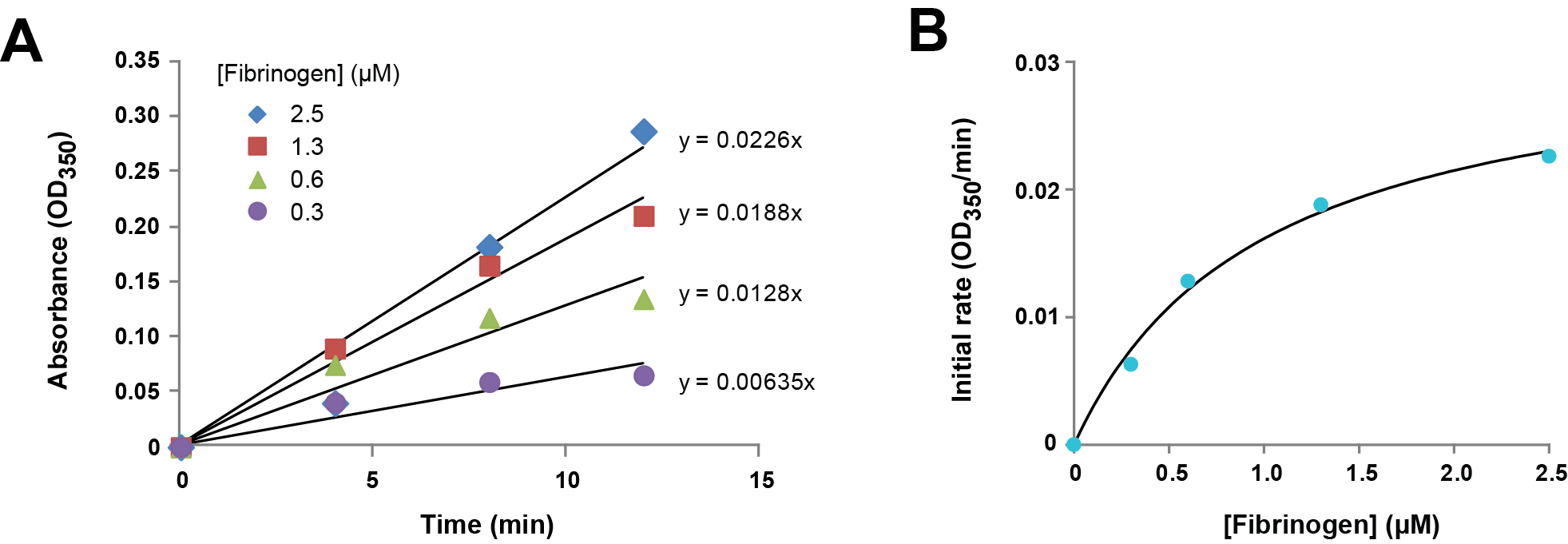


**Figure S5.** (A) Measurement of initial rates of thrombin catalysis. To determine the K_m_ for the thrombin-catalyzed conversion of fibrinogen into fibrin, we measured the initial reaction rate over a range of fibrinogen concentrations with 1 nM thrombin. (B) A nonlinear fit of the initial reaction rates plotted against the fibrinogen concentration yields a K_m_ of 0.988 µM.


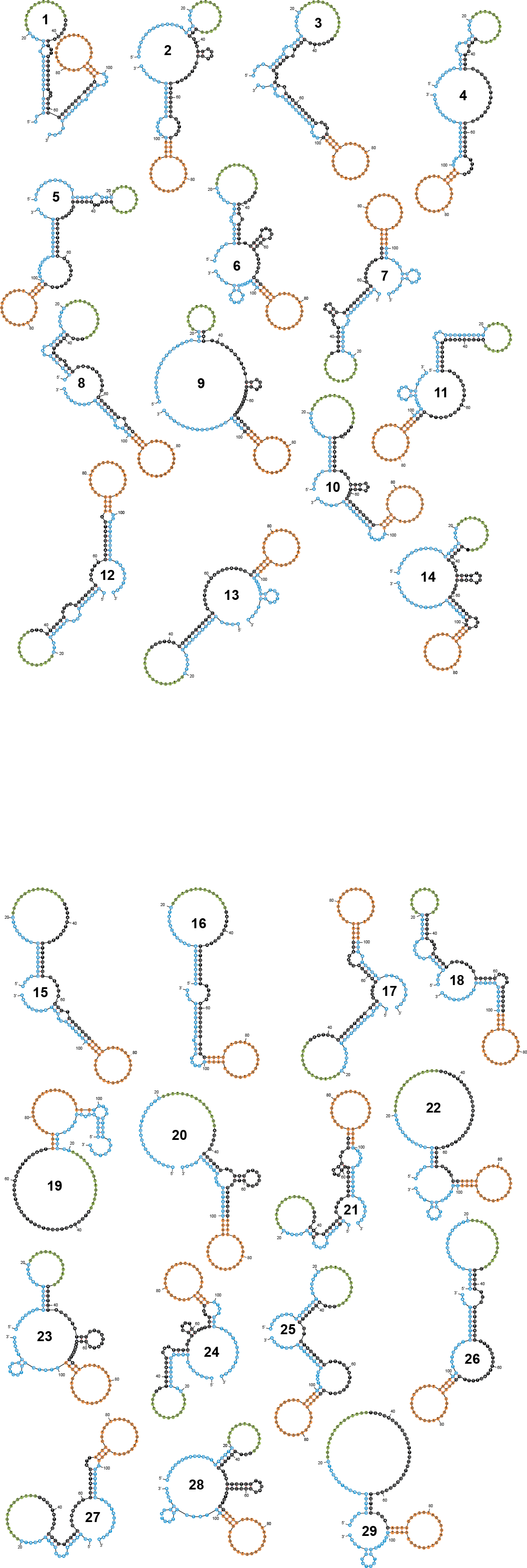


**
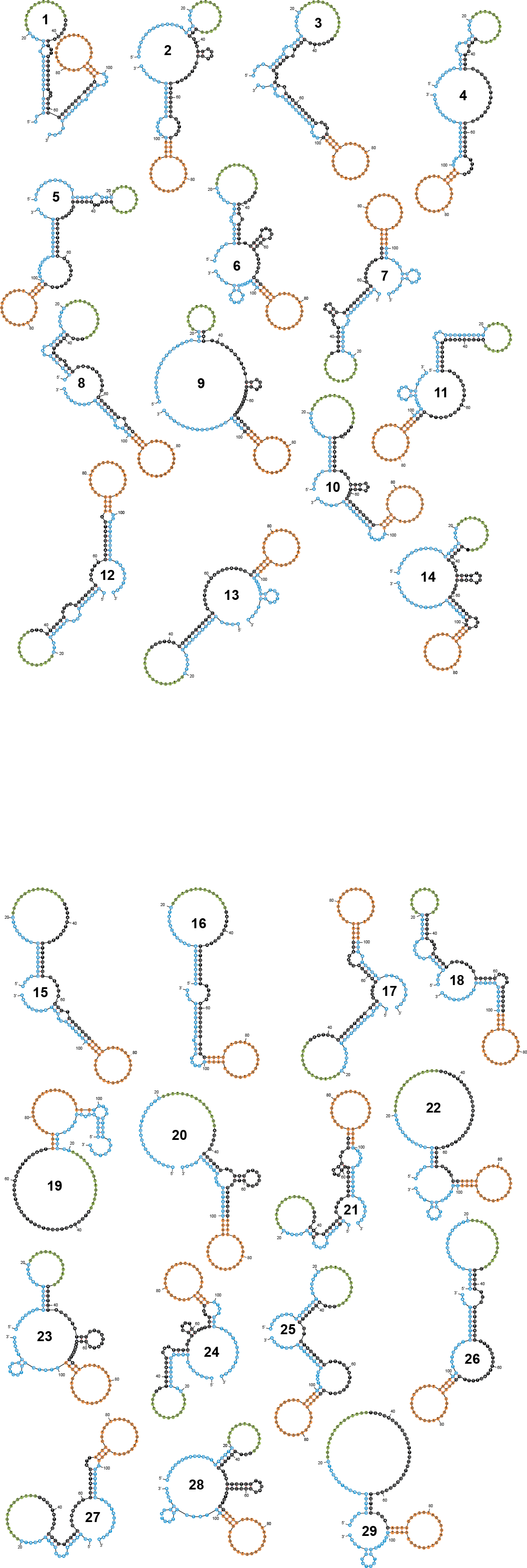
**

**Figure S6.** Secondary structures of 29 selected sequences. The lowest energy secondary structures predicted by mfold are shown with the parental aptamers highlighted in green (Bock-15) and orange (Tasset-29), the primer sites in blue, and the selected region in black. Although all sequences were unique, they show many common structural features.


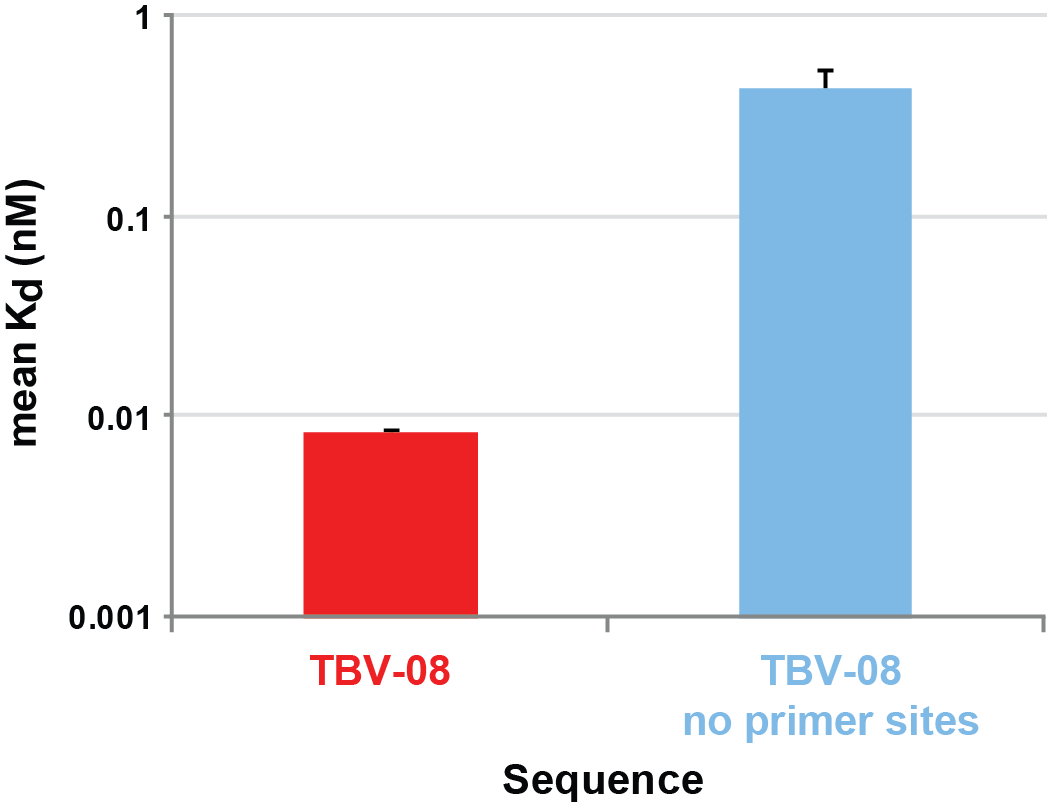


**Figure S7.** Removing the primer sites from TBV-08 substantially lowered the apparent affinity of the aptamer from 8.1 to 430 pM.
