# Supplementary material for: Selection is more intelligent than design: improving the affinity of a bivalent ligand through directed evolution
Source: Nucleic Acids Res. 2012 Oct 5;40(22):11777–83. doi: 10.1093/nar/gks899 (PMC3526301; doi:10.1093/nar/gks899)
Supplement: Supplementary Data [file supp_40_22_11777_v2_index.html]

Selection is more intelligent than design: improving the affinity of a bivalent ligand through directed evolution — Supplementary Data 

# Selection is more intelligent than design: improving the affinity of a bivalent ligand through directed evolution

## Supplementary Data

files

**Files in this Data Supplement:**

- Supplementary Data - docx file
